# Blinded trial of multiplex serodiagnostic test in India for diverse forms of active tuberculosis

**DOI:** 10.1128/spectrum.03898-25

**Published:** 2026-05-26

**Authors:** Ritu Singhal, Resmi Ravindran, Sanket Poniya, Lokender Kumar, Vithal P. Myneedu, Rohit Sarin, Pankaj Krishna, Ed Goldberg, Stephen J. Dunn, Puneet Gupta, Scott Bornheimer, Imran H. Khan

**Affiliations:** 1 Department of Microbiology, National Institute of Tuberculosis and Respiratory Diseases 28858, New Delhi, India; 2 Department of Pathology and Laboratory Medicine, University of California 8789, Davis, California, USA; 3 NextGen In Vitro Diagnostics, New Delhi, India; 4 Department of TB and Chest Diseases, National Institute of Tuberculosis and Respiratory Diseases 28858, New Delhi, India; 5 OptimAES LLC, Los Gatos, California, USA; 6 AppGenex Diagnostics, Inc., Mountain View, California, USA; 7 Tropical Animal Genetics Pvt Ltd, Chennai, India; 8 Becton Dickinson, San Jose, California, USA; Naturwissenschaftliches und Medizinisches Institut an der Universitat Tubingen, Reutlingen, Germany

**Keywords:** serodiagnosis, tuberculosis, biomarkers, antibody profiling

## Abstract

Rapid, high-throughput, and point-of-care tests to detect diverse major forms of active tuberculosis (TB) are urgently needed. Our blood test can fulfill that need. The blinded trial of the multiplex serodiagnostic test in India described here builds on our previously published field studies in diverse endemic regions (India, Uganda, and Pakistan). Antibody responses to *Mycobacterium tuberculosis* (*M. tb*.) were profiled in blood plasma using a multiplex test that comprised 12 *M. tb*. antigens. The samples included TB patients with adult pulmonary TB (APTB; including clinically diagnosed cases), extrapulmonary TB (EPTB), pediatric TB (PEDTB), and TB in HIV-positive individuals. Control groups included healthy individuals and lung disease patients, where TB was ruled out by liquid culture (disease controls). The results demonstrated the utility of our test across diverse forms of TB: microbiologically confirmed APTB, clinically diagnosed TB, PEDTB, and EPTB, with sensitivities of 90.8%, 52.2%, 87.0%, and 64.5%, respectively. The test demonstrated good specificity in control groups (healthy 90.3% and disease controls 78.4%). This non-sputum-based test detects diverse forms of TB with sensitivity and specificity better than the WHO Target Product Profile for a triage test. It is technology-platform agnostic and amenable to high-throughput and point-of-care applications.

**IMPORTANCE:**

Sputum-based tests for tuberculosis (TB) have important limitations: smear microscopy has poor sensitivity; liquid culture, although the gold standard, is slow and only about 85% sensitive; and rapid molecular assays still rely on sputum. These limitations are especially pronounced in children, patients with extrapulmonary TB, HIV-TB, and individuals unable to produce sputum. In a blinded trial in India, we evaluated a simple blood test that detects antibodies to multiple *Mycobacterium tuberculosis* proteins. The test demonstrated high sensitivity across diverse forms of active TB, including adult pulmonary TB, extrapulmonary TB, pediatric TB, HIV-TB, and microbiologically negative TB, with good specificity in controls. With results available in approximately 2 h and no need to handle infectious sputum, this test demonstrates potential utility as a non-sputum, blood-based triage test in settings where sputum-based methods are inadequate.

## INTRODUCTION

About 2 billion people are infected with *Mycobacterium tuberculosis* (*M. tb*.) globally. There are about 10 million new tuberculosis (TB) cases per year. TB kills approximately 1.5 million people annually (1, 2). India alone accounts for about 25% of global cases and deaths, making it the world’s highest TB-burden country (1, 3). Most TB-endemic countries have limited resources, and high disease burden places a significant strain on healthcare systems, impacting social and economic conditions, predominantly affecting individuals in their productive years (ages 15–44) (4).

This study is supported by a strong foundation demonstrated in our previous publications, where we demonstrated the multiplex serodiagnostic test using diverse and geographically distinct TB patient populations. Our prior field studies, conducted using samples from India and Uganda (blinded) and Pakistan (unblinded), have demonstrated the diagnostic utility of multiplex antibody-based detection (5–7). This blinded trial builds on the previous findings conducted in India in well-characterized patient categories, including microbiologically confirmed and clinically confirmed (microbiologically negative) adult pulmonary TB (APTB), extrapulmonary TB (EPTB), pediatric TB (PEDTB), and HIV-TB.

TB is generally curable when diagnosed and treated in a timely manner. However, gaps in TB diagnosis continue to be a major limitation in controlling TB (8). Even the gold standard, liquid culture system (MGIT960), is only 80%–86% sensitive and is not commonly a part of the diagnostic work-up in endemic countries because it may take several weeks to yield results (9, 10). Microscopy using the Ziehl-Neelsen (ZN) staining method remains a widely used approach for primary diagnosis of TB, but its poor sensitivity (range 20%–60%) is a huge limiting factor, especially in paucibacillary EPTB, HIV-TB, and PEDTB (11–14). Modern molecular diagnostic tools like GeneXpert (Xpert), Truenat MTB assay, and Line Probe Assay (LPA) are relatively efficient and therefore have been recommended by the WHO (1). However, all molecular diagnostic methods are benchmarked against the same gold standard (liquid culture) and thus inherit the same performance limitations. Reported sensitivities, based on comparisons with Mycobacteria Growth Indicator Tube (MGIT960), include approximately 88% for GeneXpert, 85% for the Truenat MTB assay, and 80% for LPA (15–17).

Moreover, all sputum-based diagnostic tests have serious limitations for diagnosing EPTB and PEDTB, where sputum samples are either of limited use or difficult to obtain (18, 19). Additionally, handling sputum poses major biohazard risks, requiring specialized respiratory biosafety precautions (20). Furthermore, infections with non-tuberculous mycobacteria (NTM) can complicate diagnosis by mimicking *M. tb*. in smear microscopy or culture, contributing to false positives (21). Thus, sputum-based diagnostics not only suffer from limited sensitivity but are also vulnerable to specificity issues in high-burden settings.

Non-sputum specimens have the potential to circumvent the above limitations. Serology-based approaches are cost-effective for screening large populations, and serum-based samples avoid handling of the respiratory hazardous sputum samples (5, 7, 22–24). Importantly, blood-based tests offer an advantage for detecting most forms of TB, including those requiring invasive sample collection (i.e., biopsy or body fluid aspirates for EPTB, and gastric lavage for PEDTB, or paucibacillary pulmonary TB) (18, 19).

The accuracy of serological tests can vary based on test quality and antigen selection (6, 22, 25, 26). Although the WHO has given a negative recommendation for presently available serological TB tests, they have encouraged the development of improved methods for non-sputum-based TB diagnostics, including serological tests, when properly validated through blinded trials (1).

Since not all TB patients produce antibodies to the same *M. tb*. antigens, multiplex serodiagnostic tests enable the simultaneous detection of antibodies to multiple *M. tb*. antigens, capturing immune responses across diverse groups of active TB patients (5–7). Therefore, present research has focused on developing highly efficient serodiagnostic tests, using a multiplex panel of 12 *M. tb*. antigens selected for diagnostic coverage of active TB (excluding latent tuberculosis infection [LTBI]), with emphasis on sensitivity and specificity (5–7, 23, 24). Here, we report the results of a blinded trial for a multiplex serodiagnostic test designed to detect active TB, excluding LTBI and/or Bacille Calmette-Guérin (BCG) vaccination, but including APTB (microbiologically confirmed and unconfirmed), EPTB, and PEDTB.

## MATERIALS AND METHODS

### Study setting

National Institute of Tuberculosis and Respiratory Diseases (NITRD) is a tertiary-level respiratory specialty institute catering to referral TB cases from Northern India, also having 500 beds for inpatient care. The Microbiology Department at NITRD is a National Reference Laboratory for TB with a facility for all WHO-approved technologies for TB diagnostics.

### Study design

#### TB patients

At NITRD, TB patients were prospectively enrolled between October 2019 and December 2021 and diagnosed as APTB, EPTB, PEDTB, and HIV-TB based on clinical signs and symptoms, chest X-ray, and pathological and microbiological laboratory tests, as per Indian National Tuberculosis Elimination Program (NTEP) guidelines (Table 1) (27).

**TABLE 1:** Sensitivity and specificity of the multiplex serodiagnostic test^*a*^

Category	Number of samples tested	Sensitivity	Number of positive samples	Specificity	Number of negative samples
S^+^C^+^X^+^ TB	81	93.8%	76	–^*e*^	5
S^-^C^+^X^+^ TB	49	85.7%	42	–	7
S^+^C^+^X^+^ and S^−^C^+^X^+^ TB	130	90.8%	118	–	12
S^−^C^−^X^−^ TB^*c*^^,*d*^	23	52.2%	12	–	11
PEDTB^*b*^^,*d*^	23	87.0%	20	–	3
EPTB	62	64.5%	40	–	22
Clinically diagnosed EPTB^*d*^	0	–	--	–	–
HIV-TB (S^+^C^+^X^+^& S^−^C^−^X^−^^*d*^)	24	75.0%	18	–	6
Disease controls	74	–	16	78.4%	58
Healthy control IGRA^+^	26	–	3	88.5%	23
Healthy control IGRA^−^	46	–	4	91.3%	42
All healthy control (IGRA^+^ and IGRA^−^)	72	–	7	90.3%	65
Total	408	–	–	–	–

^*a*^ S^+^C^+^X^+^, Smear/culture/Xpert-positive; S^−^C^+^X^+^, Smear-negative/culture/Xpert-positive; S^−^C^−^X^−^, Smear-negative/culture/Xpert-negative; PEDTB, pediatric TB; EPTB, extrapulmonary TB.

^*b*^ PEDTB patients include both pulmonary and EPTB.

^*c*^ S^−^C^−^X^− ^group failed to respond to broad-spectrum antibiotics but successfully completed a full course of anti-tuberculosis therapy (ATT), with clinical/radiologic improvement by the end of 6 months. For the rest of the study groups, exclusion criteria included patients undergoing ATT for more than 1 month, those taking immunosuppressive agents, individuals with autoimmune diseases (excluding HIV), critically ill patients, and pregnant women.

^*d*^ The shortfall in sample size was primarily noted among microbiologically negative categories, mainly due to challenges in following up with these patients and confirming clinical tuberculosis (TB).

^*e*^ –, not applicable.

#### Disease controls

This group consisted of patients diagnosed with the following diseases (*n* = 74): interstitial lung disease (ILD), lung cancer, coronavirus disease 2019 (COVID-19), bacterial pneumonia, chronic obstructive pulmonary disease (COPD), asthma, etc., who were found to be negative for TB by liquid culture (MGIT960) and Xpert (Table 1) (6).

#### Healthy controls

Healthy controls were individuals with no known illnesses, recruited from the same geographic region as TB patients (6). Interferon-gamma release assays (IGRAs) using Quantiferon TB Gold assay were performed on all participants according to the manufacturer’s instructions (Qiagen N.V., Venlo, Netherlands). A mixture of IGRA^+^ and IGRA^−^ subjects was used to establish assay cut-off values (see below). Specificity for healthy subjects was calculated using an independent set of healthy controls (Hold-Out Set; *n* = 72; IGRA^+^ = 26, IGRA^−^ = 46) collected at a different time point and not included in the determination of the cut-off.

### Sputum sample processing and standard microbiological diagnostic procedures

Sputum samples were subjected to smear microscopy with ZN staining, and positives were graded per NTEP guidelines (28). Subsequently, each sputum sample was divided into two portions. The first sample was processed by NALC-NaOH decontamination as described earlier (29). The deposits were inoculated in MGIT 960 (BACTEC culture system) as described earlier (30). Tubes with positive alerts were confirmed for *M. tb*. by smear microscopy for serpentine cording and a rapid immunochromatographic test for detection of MPT64 TB Ag (SD BIOLINE). The Xpert MTB/RIF test (Cepheid) was performed on the second portion of the sample as per the manufacturer’s instructions.

### Blood sample collection, processing, and storage

A 5 mL blood sample was drawn by venipuncture into an EDTA Vacutainer tube. Plasma was separated by centrifugation, and multiple aliquots were frozen at –80°C as previously described (31).

### Multiplex serodiagnostic test

Recombinant *M. tb*. antigens were expressed in *Escherichia coli*, purified, and coupled to microbeads as previously described (23, 31). A multiplex microbead immunoassay based on the xMAP technology platform (Luminex Corp, Austin, TX) was performed to detect the plasma IgG antibodies using microbeads conjugated with 12 *M. tb*. antigens that were previously selected based on high sensitivity and specificity: Rv3881c, Rv0934 (P38 or PstS1), Rv2031c (HspX), Rv1886c (Ag85b), Rv1860 (MPT32), Rv3874 (CFP10), Rv1926c, Rv1984c (CFP21), Rv3841 (Bfrb1), Rv2875 (MPT70), Rv3619, and Rv0054 (7, 23, 32). In brief, a mixture of microbead sets (one for each coated antigen described above) was incubated with plasma samples diluted 1:200 in the assay diluent (2% Prionex [bio-WORLD, Dublin, OH] prepared in PBS with 0.1% Tween-20) for 1 h at room temperature on a shaker at 500 RPM in the dark. Blank, positive control, and negative control were run in duplicates on all the plates; test samples were run in single wells, with every 10th sample run in duplicate to monitor for random variation. After incubation, the liquid was removed using a vacuum manifold designed for 96-well plates (Millipore Corporation, Bedford, MA). Beads were washed twice by adding 100 μL wash buffer (PBS with 0.1% Tween-20) per well and drained under vacuum. For the detection of human IgG, phycoerythrin conjugated anti-human IgG (Jackson ImmunoResearch, West Grove, PA) was used at a 1:500 dilution in wash buffer; 50 μL was added per well, and plates were incubated for 15 min at room temperature on a shaker at 500 RPM in the dark. Beads were then washed twice, resuspended in 100 μL wash buffer per well, and analyzed on a MAGPIX instrument (Luminex Corp, Austin, Texas) using xPONENT software v4.2, acquiring a minimum of 50 beads per region, per well. The data were reported as median fluorescence intensities (MFI), as previously described (6).

Assay reproducibility, limits of blanks, and assay cut-offs have been evaluated in our prior publications on field trials on TB patients in India, Pakistan, and Uganda, as well as in animal models of TB (non-human primates and rabbits) (5–7, 23, 32–34). Each plate was set up as described above. Blank wells received beads, assay diluent, and secondary antibody. Positive control consisted of a pooled mixture of plasma samples from five TB patients selected to provide antibody reactivity above antigen-specific cut-off values across the panel of antigen-coupled beads (the exception was Rv3619, for which the only TB patient plasma sample available in sufficient quantity to include as a control across all plates for the entire study was a low-positive sample). Negative control consisted of a pooled mixture of plasma from 10 healthy individuals. Six plates were run to cover the entire study population. All the plates were run on different days. Plate 1 was the first, and the other five were run in the order reflected by their numbers. Blank and control results, as well as intra- and interassay coefficients of variation (CVs) (%), are provided in Table S4. Intraassay CVs (%) were calculated from the duplicate positive-control wells on each plate. Interassay CVs (%) were calculated using duplicate positive-control wells across all six plates.

### Statistical analysis

To account for local exposure patterns, including BCG vaccination and NTM, cut-off values were calculated for each antigen as Mean MFI + (3 × SD) using IGRA^+^ and IGRA^−^ healthy controls recruited from the same endemic region as the TB cases. These cut-offs were then applied to all TB and control samples. To evaluate specificity in healthy controls, an independent Hold-Out Set was analyzed (described in Study Design, *Healthy Controls*). Samples with antibodies against one or more of the 12 antigens, above the cut-off, were considered positive. Cut-off values for each antigen are provided in Table S2.

## RESULTS

### Antibody profiles in different groups of patients and healthy controls

**Fig 1:**
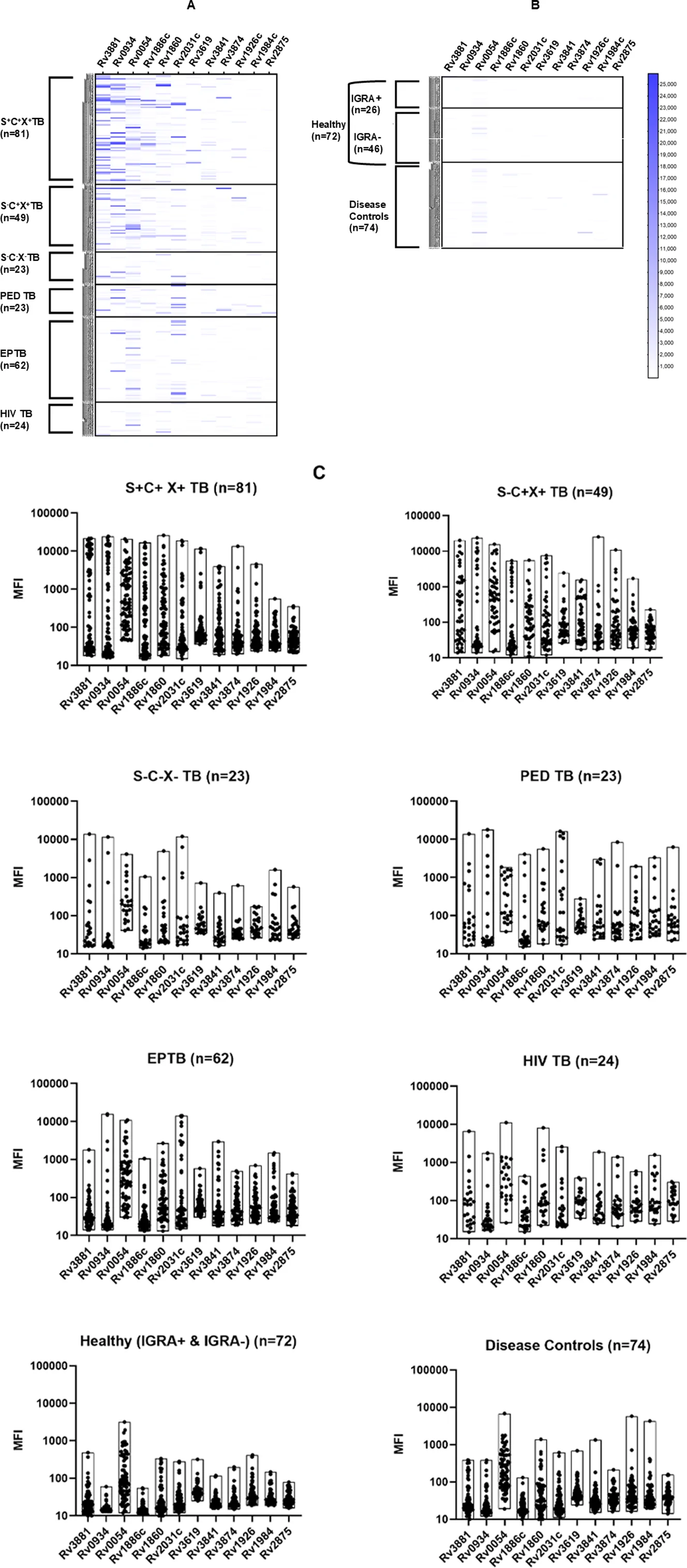
Raw MFI data to visualize anti-*M. tb.* antibody responses detected by the multiplex serodiagnostic test across various sample groups: Panels** A** and **B** show heatmaps for TB patients and Controls (Healthy and Disease Controls), respectively. Antigens shown at the top of the two panels are arranged in descending order (left to right) based on overall antibody prevalence in the microbiologically confirmed adult pulmonary TB groups (S^+^C^+^X^+^ and S^−^C^+^X^+^). Samples are arranged horizontally and antibodies vertically. The heatmap was generated using GraphPad Prism v10.2.2. Color intensity indicates (MFI), ranging from high MFI (red, 26,000 MFI) to baseline MFI (black, 0 MFI). (**C**) Boxplots showing raw MFI values for each antigen across study groups with individual data points. The Y-axis scale is consistent across all plots to facilitate direct comparison between groups. Cut-off values for reference, for each antigen, are provided in Tables S2 and S4. The following abbreviations are used: MFI, median fluorescence intensity; S^+^C^+^X^+^, Smear/culture/Xpert-positive; S^−^C^+^X^+^, Smear-negative/culture/Xpert-positive; S^−^C^−^X^−^, Smear-negative/culture/Xpert-negative; PEDTB, pediatric TB; EPTB, extrapulmonary TB.

Antibodies against selected 12 *M. tb*. antigens in multiplex format were profiled by the multiplex serodiagnostic test (Fig. 1). The two microbiologically confirmed groups, smear positive, culture positive, Gene Xpert positive (S^+^C^+^X^+^; *n* = 81) and smear negative, culture positive, Gene Xpert positive (S^−^C^+^X^+^; *n* = 49), are shown in the top panel (A). These two groups displayed similar antibody profiles. The highest prevalence of antibodies is against the following six antigens: Rv3881, Rv0934, Rv0054, Rv1886c, Rv1860, and Rv2031, as we have previously described in TB patients in our previous studies (6, 7). Antibodies against other antigens were less prevalent but proved valuable in improving sensitivity since individual antigens demonstrated additive effects (Table 3). The clinically diagnosed (smear negative, culture negative, and Gene Xpert negative [S^−^C^−^X^−^]) group exhibited a lower number of positive patients and MFI intensities. The PEDTB and EPTB groups exhibited profiles similar to each other but different from the adult pulmonary TB; Rv2031 showed the highest prevalence. The HIV-TB group showed antibody responses similar to the S^−^C^−^X^−^ group. Healthy controls (IGRA^+^ and IGRA^−^) exhibited barely detectable false positive signal (B). Among the disease controls, as in IGRA^−^ healthy controls, low-level MFIs were observed against Rv0054 in a few samples. The heatmap displays raw MFI values for visualization only; the color scale represents the relative strength of antibody reactivity and shows background reactivity in healthy and disease control groups. Because the figure is not quantitative, apparent differences in MFI intensity between groups cannot be interpreted. For a direct view of the antibody reactivity within each group for each antigen, box plots show raw MFI values across study groups with individual data points (Fig. 1C). Diagnostic classification relied on antigen cut-offs (Table S2). Raw MFI values are provided in Table S3. Intra- and interassay CVs (%) are provided (Table S4). The interassay CV for Rv1886c exceeded 15%.

**Fig 2:**
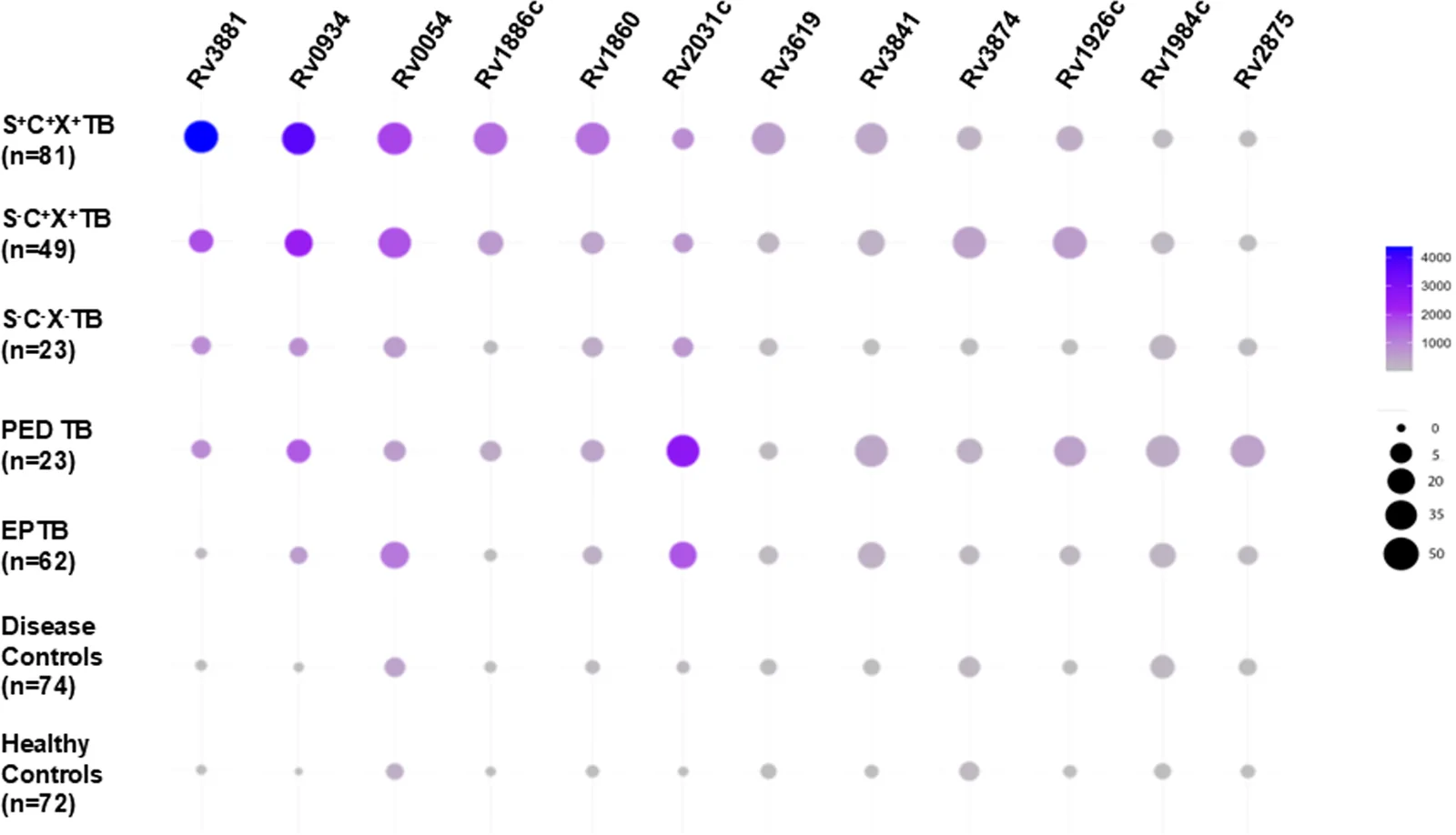
Raw MFI data depicting antibody responses detected by the multiplex serodiagnostic test across subject groups. Average MFI and the percentage of antibody-positive samples against *M. tb.* antigens are shown. Antigens are arranged at the top as in Fig. 1A and B. The color scale represents MFI intensity averaged for each group (blue = high, gray = low), and the circle size represents the percentage of antibody-positive subjects within each group. Data are shown for adult pulmonary TB (S^+^C^+^X^+^, S^−^C^+^X^+^, and S^−^C^−^X^−^), PEDTB, EPTB, disease controls, and healthy controls. Analysis was performed using R, version 3.0.2 (R Foundation, Vienna, Austria). The following abbreviations are used: MFI, median fluorescence intensity; S^+^C^+^X^+^, Smear/culture/Xpert-positive; S^−^C^+^X^+^, Smear-negative/culture/Xpert-positive; S^−^C^−^X^−^, Smear-negative/culture/Xpert-negative; PEDTB, pediatric TB; EPTB, extrapulmonary TB.

For an easy and direct comparison of antibody profiles between subject groups, a dot plot analysis is presented (Fig. 2). It shows the advantage of relative prevalence and intensity of antibodies for a quick comparison between different groups. For example, it can be visualized how similar the overall profiles in S^+^C^+^X^+^ and S^−^C^+^X^+^ are and how different they are from EPTB and PEDTB. In the latter two groups, the most prominent antibodies are against Rv2031c compared to adult pulmonary TB. Similarly, the profiles in the two control groups (Healthy and Disease Controls) are more similar to each other than to any other group. Figure 2 is a descriptive visualization of raw data and shows background reactivity in healthy and disease control groups; test results are not interpreted from dot sizes or colors.

### Performance of the multiplex serodiagnostic test

The sensitivity and specificity of the multiplex test were calculated using cut-offs determined for each antigen in the Healthy Controls (Table 1).

#### Sensitivity and specificity

The sensitivities of the multiplex antibody test for the two microbiologically confirmed adult pulmonary TB groups were as follows: 93.8% for S^+^C^+^X^+^ group, and 85.7% for smear negative, but culture and Xpert positive (S^−^C^+^X^+^) group. For these two groups combined (S^+^C^+^X^+^ and S^−^C^+^X^+^), accounting for all microbiologically confirmed adult pulmonary TB patients, the sensitivity was 90.8%.

For the patient groups not detected by the gold-standard diagnostic test, the sensitivities were as follows: for the clinically diagnosed group (S^−^C^−^X^−^; microbiologically unconfirmed), sensitivity was 52.2%, for PEDTB, it was 87.0%, and for EPTB, it was 64.5%.

For HIV positives with both microbiologically and clinically confirmed TB, the combined sensitivity was 75.0%.

The specificity for healthy individuals was 90.3%, with no notable difference between IGRA^+^ (88.5%) or IGRA^−^ (91.3%) healthy controls, while the specificity for the disease control group was 78.4%. In principle, Disease Controls and healthy populations should have similar specificities because the likelihood of non-specific humoral immune responses is primarily due to infections by NTM and other environmental factors. Since healthy subjects are from the same local geographic region, their exposure is the same as that of the Disease Control patients. Therefore, the lower specificity observed in the Disease Control group strongly suggests the possibility of false-negative results by the MGIT culture system (gold standard). MGIT is well documented to yield 13.5%–20.0% false negatives in TB patients (9, 10).

The test demonstrated high PPV (94.4%) and NPV (84.4%), with an overall accuracy of 90.6% in all culture and GeneXpert-positive adult pulmonary patients, which represent an overwhelming majority of TB patients (Table 2). A comparison of the multiplex serodiagnostic test with the published performance of GeneXpert and Truenat is presented (Table S1) (35, 36).

**TABLE 2:** Performance measures of the multiplex serodiagnostic test^*a*^

Parameters	Based on healthy controls		Based on disease controls	
	Value	95% CI	Value	95% CI
PPV	94.4%	88.9%–97.3%	88.6%	81.5%–92.5%
NPV	84.4%	74.7%–90.9%	82.9%	72.4%–89.9%
LR^+^	9.34	4.61–18.91	4.20	2.71–6.50
LR^−^	0.10	0.06–0.18	0.12	0.07–0.20
Accuracy	90.6%	85.8%–93.9%	86.3%	80.9%–90.3%

^*a*^ Statistical parameters were calculated using data from all culture and GeneXpert positive adult pulmonary TB cases (S^+^C^+^X^+^ and S^−^C^+^X^+^), which represent an overwhelming majority of the cases in a given TB patient population. PPV, positive predictive value; NPV, negative predictive value; LR^+^, positive likelihood ratio; LR^−^, negative likelihood ratio.

### Additive effects of individual antigens on the performance of multiplex serodiagnostic test

The additive effect of individual *M. tb*. antigens on the sensitivity and specificity of the multiplex serodiagnostic test was analyzed using a sequential addition approach in the calculations (Table 3). Antigens were added one at a time to see the effect, in ascending order of the proportion of antibody-positive TB samples. As an example, the best sensitivity for a single antigen is for Rv1886c in the patient group S^+^C^+^X^+^; sensitivity for this group improves dramatically as additional antigens are included in the calculations.

**TABLE 3:** Effect of sequential single antigen addition^*a*^

Groups	Rv1886c	Rv3881	Rv0934	Rv3841	Rv0054	Rv2031c	Rv1860	Rv3874	Rv1926c	Rv3619	Rv1984	Rv2875
	Sensitivity (%)											
S^+^C^+^X^+^	48.1	69.1	76.5	79.0	85.2	86.4	90.1	90.1	91.4	93.8	93.8	93.8
S^−^C^+^X^+^	30.0	54.0	60.0	74.0	80.0	82.0	84.0	86.0	86.0	86.0	86.0	86.0
S^+^C^+^X^+^ and S^−^C^+^X^+^	40.3	62.6	69.4	76.9	83.0	84.5	87.5	88.3	89.0	90.5	90.5	90.5
S^−^C^−^X^−^	17.4	30.4	34.8	34.8	39.1	43.5	47.8	47.8	47.8	47.8	47.8	52.2
PEDTB	26.1	43.5	47.8	60.9	69.6	73.9	73.9	78.3	78.3	78.3	87.0	87.0
EPTB	6.5	8.1	14.5	29.0	38.7	50.0	51.6	61.3	62.9	62.9	62.9	64.5
HIV-TB	20.8	29.2	37.5	45.8	54.2	58.3	58.3	58.3	66.7	66.7	75.0	75.0
	Specificity (%)											
Disease controls	97.3	97.3	95.9	93.2	83.8	82.4	81.1	79.7	79.7	79.7	78.4	78.4
Healthy	100.0	98.6	98.6	98.6	94.4	94.4	94.4	91.7	90.3	90.3	90.3	90.3

^*a*^ S^+^C^+^X^+^, Smear/culture/Xpert-positive; S^−^C^+^X^+^, Smear-negative/culture/Xpert-positive; S^−^C^−^X^−^, Smear-negative/culture/Xpert-negative; PEDTB, pediatric TB; EPTB, extrapulmonary TB. Each column represents the test performance with the cumulative inclusion of an additional antigen.

## DISCUSSION

The results of the blinded trial demonstrated that the multiplex serology test satisfies the WHO’s target product profile (TPP) criteria for a TB triage test that suggests 90% sensitivity in adult pulmonary TB and 70% specificity (37). In this trial, for the two adult pulmonary TB groups (S^−^C^+^X^+^ and S^+^C^+^X^+^), which are culture and GeneXpert-positive and represent an overwhelming majority of cases in a given TB patient population, the respective sensitivities were 93.8% and 85.7%, and the overall sensitivity for the two groups together was 90.8%. The specificity for healthy controls is 90.3% and for Disease Controls is 78.4%. The test demonstrates potential utility in patient groups not detected by the gold-standard diagnostic test (liquid culture), such as S^–^C^–^X^–^ (microbiologically negative adult pulmonary TB), EPTB, and PEDTB (Table 1). The respective sensitivities for these groups were 52.2%, 64.5%, and 87.0%. We have combined pediatric pulmonary and extrapulmonary TB in a single category, which may provide different results.

Test specificity among healthy controls (combined IGRA^+^ and IGRA^−^) was high at 90.3%, with negligible difference between IGRA^+^ and IGRA^−^ subgroups. In TB endemic countries, healthy individuals are frequently exposed to *M. tb*. and carry latent infection (36% in India and 25% globally), resulting in IGRA positivity (38, 39). This is reflected by the IGRA results among the healthy controls in this study, 26 IGRA positives out of 72 (Table 1). However, since our multiplex serodiagnostic test only includes *M. tb*. antigens that have low non-specific antibody reactivity, regardless of IGRA test results in healthy individuals, it suggests that the performance of the multiplex serology test is not affected in a significant way by LTBI (6, 7). Specificity of 88.5% in IGRA^+^ healthy controls (Table 1) supports the validity of using healthy controls from TB-endemic regions to establish cut-offs. Although specificity among healthy endemic controls was high, our study did not include an NTM disease control group; therefore, specificity relative to NTM disease requires further validation. Because cut-offs were derived from the healthy controls from the same endemic region as the patients, these cut-offs intrinsically account for the background reactivity of the antigens to NTM.

Specificity among disease controls was lower, at 78.4%. In principle, specificity in the Disease Control group should be close to that of healthy controls, as both populations are likely to have similar environmental exposure to NTM as well as other environmental immunogens and factors, leading to non-specific humoral immune responses (40). Nonetheless, lower specificity observed in Disease Controls is possibly due to the presence of co-existent low-level or incipient TB that could not be diagnosed because of the lack of a classical clinical picture and/or radiological findings and was also undetected by MGIT. The MGIT960 culture is known to have sensitivity ranging from 80% to 86%, as demonstrated in the significant proportion of pulmonary TB cases turning up false negatives in published studies (9, 10). Therefore, such false negative samples are highly likely to be registered as positives by our multiplex serology test, thereby leading to low specificity of the Disease Control group compared to healthy subjects.

The specificity of a diagnostic test can be substantially underestimated when evaluated against an imperfect gold standard, particularly in TB hyperendemic settings, since true positives missed by the gold standard may be misclassified as false positives (41, 42). It is important to note that our multiplex antibody test detected 52.2% of clinically diagnosed (S^−^C^−^X^−^) cases that were negative by culture (MGIT960) and Xpert (in addition to being smear negative). These patients failed to respond to broad-spectrum antibiotics but successfully completed a full course of anti-tuberculosis therapy, with clinical/radiological improvement by the end of 6 months. Although microbiological confirmation was unavailable, these data suggest that these patients may be TB cases undetected by MGIT960. Therefore, the positive results from the multiplex serology test in this group likely represent true positives rather than false positives. Thus, they reinforce the diagnostic capabilities of the multiplex antibody test in detecting TB cases that are missed by MGIT960 and Xpert.

The above differences may also highlight the drastic biological differences in the natures of the two types of specimens—blood versus sputum—each with its own advantages and disadvantages.

Furthermore, in TB-hyperendemic settings, as observed in the present trial, other dominant diseases (e.g., COPD, lung cancer, etc.) may mask the underlying TB. The risk of developing active TB varies across chronic lung diseases but is high because of the following diseases: individuals with COPD have an approximately fourfold increased risk, those with a history of pneumonia approximately 3.4-fold, asthma a 1.37-fold, lung cancer a 1.64- to 6.0-fold, and ILD, particularly silicosis, a 1.44- to 3.14-fold increased risk compared to individuals without these conditions. Hence, some individuals classified as Disease Controls harbored undetected or incipient TB, not detected by the sputum-based MGIT960 but were detected by the blood-based multiplex serology test, thereby contributing to the reduced specificity observed in the Disease Control group (43).

In our prior publications, we have demonstrated consistent performance of our multiplex serology test across independent cohorts in different field studies in multiple TB-endemic settings, including blinded studies in Uganda and India (5, 6) and unblinded studies in Pakistan (6, 7, 32). Our studies are also consistent with the results of a comprehensive genome-wide analysis that identified *M. tb*. antigens of high importance for TB serodiagnosis (44). Other groups have used similar antigens on the Luminex platform and reported different performance in their study population (45). The differences may be due to a whole host of factors, such as protein (antigen) purity, protein conformation, availability of certain epitopes after cross-linking to the microbeads, optimization of protein concentration, optimization of cross-linking, other probable antigen-related preparatory issues, and optimization of assay conditions.

This study has a few limitations. First, we have combined pediatric pulmonary and extrapulmonary TB in a single category, which may confound the results. Second, although specificity among healthy endemic controls was high, our study did not include NTM disease control cohorts; specificity relative to NTM disease requires further validation.

Given that the test performance satisfies the WHO’s TPP criteria for a triage test, the results presented here support the potential utility of the multiplex serology test for triaging (37). The present trial analyzed samples from several forms of TB and demonstrated that incorporating multiple *M. tb*. antigens enhances diagnostic sensitivity in these groups (Fig. 1 and 2).

Our multiplex serology test provides an efficient alternative to conventional TB diagnostics, including sputum smear microscopy, culture, and WHO-approved real-time PCR tests such as Xpert MTB/RIF and Truenat MTB (Table S1), particularly in cases where sputum is difficult to obtain and yields low sensitivity, such as EPTB, PEDTB, smear-negative cases, and HIV-TB (Table S1) (35, 36). Importantly, the test does not require biosafety level 3 respiratory pathogen containment. The feasibility of using dried blood spots (DBS), collected via simple finger prick onto filter paper, for this multiplex serodiagnostic test was explored in preliminary laboratory work, and the test appears compatible with DBS eluates (unpublished data, Khan, I.H., UC Davis). This minimally invasive method is ideal for remote or rural settings, as DBS cards can be easily transported to testing centers. However, DBS was not evaluated in this study, and the implementation will require further validation.

From a clinical workflow perspective, the test provides results in 2 h and can process up to 360 samples (a single 96-well plate) within an 8 h day in a clinical laboratory setting. It is ideal for systematic TB screening in high-burden areas, such as the Indian subcontinent (where more than a third of the world’s TB occurs), and can be adapted to various clinical settings. With readily available automation, the system can scale to handle thousands of samples daily. The test is platform agnostic and can be configured for both high-throughput (i.e., population-level screening) and, with further development, adapted for Point-of-Care platforms (i.e., individual patients in a clinical setting), supporting diverse clinical needs.

### Conclusion

The multiplex serodiagnostic test clinically validated in this blinded trial shows utility not only in detecting adult pulmonary TB but also for the detection of hard-to-diagnose forms of the disease, e.g., microbiologically negative TB, EPTB, HIV-TB, and PEDTB. This blood-based test eliminates the requirement for increased respiratory protection for technical staff; hence, it can be used in a non-microbiological clinical laboratory. This test leverages established platforms in the clinical laboratory, such as a flow cytometer, providing a possibility of integrating the test into the high-throughput diagnostic capabilities of current clinical laboratories and widespread implementation.

## SUPPLEMENTAL MATERIAL

Supplemental tables spectrum.03898-25-s0001.docx Tables S1 and S2.

Table S3 spectrum.03898-25-s0002.xlsx Raw data MFI-TB patients, healthy and disease controls.

Table S4 spectrum.03898-25-s0003.xlsx Intra and interassay CV (%) of the multiplex serodiagnostic test.

ASM does not own the copyrights to Supplemental Material that may be linked to, or accessed through, an article. The authors have granted ASM a non-exclusive, world-wide license to publish the Supplemental Material files. Please contact the corresponding author directly for reuse.
