# Supplementary material for: Blinded trial of multiplex serodiagnostic test in India for diverse forms of active tuberculosis
Source: Microbiol Spectr. 2026 May 26;14(7):e03898-25. doi: 10.1128/spectrum.03898-25 (PMC13339956; doi:10.1128/spectrum.03898-25)
Supplement: Supplemental tables — Tables S1 and S2. [file spectrum.03898-25-s0001.docx]

**Table S1** Comparison of sensitivity and specificity of the multiplex serodiagnostic test to TB molecular tests

| **Category** | **Sensitivity (%)** | | | **Specificity (%)** | | |
| --- | --- | --- | --- | --- | --- | --- |
|  | **Multiplex Test** | **Xpert MTB/RIF** | **Truenat** | **Multiplex Test** | **Xpert MTB/RIF** | **Truenat** |
| S^+^C^+^X^+^ | 93.8 | - | - | - | - | - |
| S^-^C^+^X^+^ | 85.7 | 61 | 36 | - | - | - |
| S^+^C^+^X^+^ & S^-^C^+^X^+^ | 90.8 | 85 | 73 | - | - | - |
| S^-^C^-^X^-^ | 52.2 | - | - | - | - | - |
| PEDTB | 87.0 | - | - | - | - | - |
| EPTB | 64.5 | - | - | - | - | - |
| HIV^+^S^+^C^+^X^+^ | 91.7 | 75 | - | - | - | - |
| HIV^+^S^-^C^-^X^-^ | 58.3 | - | - | - | - | - |
| Disease Controls | - | - | - | 78.4 | 98 | 98 |
| Healthy Controls | - | - | - | 90.3 | - | - |

The following abbreviations are used: S^+^C^+^X^+^ = Smear/culture/Xpert-positive; S^-^C^+^X^+^ = Smear-negative/culture/Xpert-positive; S^-^C^-^X^-^ = Smear-negative/culture/Xpert-negative; PEDTB= Pediatric TB, EPTB = Extrapulmonary TB. En dash (–) indicates not applicable or not available.

Data for Xpert MTB/RIF and Truenat were obtained from previous publications (35, 36).

**Table S2 Cut-off values for each antigen**

| **Antigens** | **Cut-off values** |
| --- | --- |
| Rv3881c | 442 |
| Rv0934 | 221 |
| Rv0054 | 1158 |
| Rv1886c | 70 |
| Rv1860 | 965 |
| Rv2031c | 348 |
| Rv3619 | 534 |
| Rv3841 | 154 |
| Rv3874 | 162 |
| Rv1926c | 390 |
| Rv1984c | 444 |
| Rv2875 | 274 |
